# Internal Doses of Glycidol in Children and Estimation of Associated Cancer Risk

**DOI:** 10.3390/toxics7010007

**Published:** 2019-02-01

**Authors:** Jenny Aasa, Efstathios Vryonidis, Lilianne Abramsson-Zetterberg, Margareta Törnqvist

**Affiliations:** 1 Department of Environmental Science and Analytical Chemistry, Stockholm University, 106 91 Stockholm, Sweden; jenny.aasa@aces.su.se (J.A.); efstathios.vryonidis@aces.su.se (E.V.); 2 National Food Agency, 751 26 Uppsala, Sweden; lilianne.abramsson@slv.se

**Keywords:** glycidol, Hb adduct, *N*-(2.3-dihydroxypropyl)valine, in vivo, cancer risk, UPLC/MS/MS

## Abstract

The general population is exposed to the genotoxic carcinogen glycidol via food containing refined edible oils where glycidol is present in the form of fatty acid esters. In this study, internal (in vivo) doses of glycidol were determined in a cohort of 50 children and in a reference group of 12 adults (non-smokers and smokers). The lifetime in vivo doses and intakes of glycidol were calculated from the levels of the hemoglobin (Hb) adduct *N*-(2,3-dihydroxypropyl)valine in blood samples from the subjects, demonstrating a fivefold variation between the children. The estimated mean intake (1.4 μg/kg/day) was about two times higher, compared to the estimated intake for children by the European Food Safety Authority. The data from adults indicate that the non-smoking and smoking subjects are exposed to about the same or higher levels compared to the children, respectively. The estimated lifetime cancer risk (200/10^5^) was calculated by a multiplicative risk model from the lifetime in vivo doses of glycidol in the children, and exceeds what is considered to be an acceptable cancer risk. The results emphasize the importance to further clarify exposure to glycidol and other possible precursors that could give a contribution to the observed adduct levels.

## 1. Introduction

Exposure to genotoxic compounds in interaction with other factors contributes to an increased risk of cancer development [1]. For many cancer types, the onset of the disease are several decades after a specific exposure [2]. Quantification of the cancer risk from exposure to genotoxic compounds is usually based on data from carcinogenicity studies at high doses in rodents, with extrapolation of the obtained cancer risk coefficient to estimated human exposure doses of the studied compound. An improved estimate of the risk would be obtained if the internal (in vivo) dose of the studied compound/metabolite could be used for species extrapolations. This is particularly important for ongoing human exposures, where measured in vivo doses also give an improved estimate of the exposure. 

**Figure 1:**
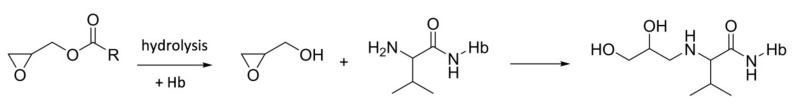
Glycidol is formed in vivo by hydrolysis of glycidyl fatty acid esters [3], where R represents different ester side chains. Glycidol reacts with the N-terminal valine in hemoglobin (Hb) to form *N*-(2,3-dihydroxypropyl)valine (diHOPrVal) as N-terminal.

One compound, for which there is an ongoing human exposure, is the genotoxic compound glycidol [3]. This compound has received much attention during the last years, largely due to the detection of glycidyl fatty acid esters in food intended for children, such as as infant formula [4]. Possible exposure sources are food products containing refined edible oils where glycidol is present in the form of glycidyl fatty acid esters [5,6]. The esters are hydrolyzed in the stomach, leading to formation of glycidol (Figure 1) [7]. This is of concern to human health, as glycidol is classified by the International Agency for Research on Cancer (IARC) as probably carcinogenic to humans, Group 2A [8]. Protecting children from exposure to genotoxic compounds is important. Children are considered more vulnerable for exposure to chemical compounds compared with adults, because children are still growing and organ cells are rapidly dividing [9]. This likely increases the rate of fixation of mutations, which can lead to the onset of cancer.

Here, we will present results from a study of children where the in vivo dose of glycidol has been quantified in blood samples from the individuals. Also, blood samples from 12 adults were analyzed as a reference group. As genotoxic chemicals are usually electrophiles and short-lived in vivo, measurement in vivo of the ultimate genotoxic compound/metabolite is generally difficult per se. Instead, stable adducts to proteins may be used as a biomarker of exposure/internal dose. We have previously developed a method for measurement of adducts to the N-terminal valine in hemoglobin (Hb) using GC/MS/MS [10]. Later, the method was developed for LC/MS/MS, and is referred to as the FIRE procedure [11,12]. If the reaction rate constant for the formation of the specific adduct from the studied electrophile to the N-terminal valine is known, the in vivo dose expressed as the area under the concentration–time curve (AUC) can be calculated for the electrophile from a measured Hb adduct level [13]. This analytical approach has previously been applied in studies of exposed animals and humans for monitoring of internal exposures and quantification of in vivo doses of reactive compounds, for instance present in food [14,15,16], or for the screening of adducts with an adductomics approach for the investigation of background exposures in humans [17,18]. In the present study, in vivo doses of glycidol were calculated from measured Hb adduct levels in the human blood samples (Figure 1). The in vivo doses were then used for the estimation of the lifetime excess cancer risk, due to glycidol exposure.

## 2. Materials and Methods

### 2.1. Chemicals

Glycidol (98%, CAS No. 556-52-5) was purchased from Acros Organics (Geel, Belgium). L-Valine-(^13^C_5_) (96–98% purity), RS-glyceraldehyde and sodium cyanoborohydride, used for the synthesis of the internal standard *N*-(2,3-dihydroxypropyl)-(^13^C_5_)valine, were obtained from Cambridge Isotope Laboratories, Inc (Tewksbury, MA, USA) and Sigma Aldrich (St Louis, MO, USA) respectively. Cyanoacetic acid and ammonium hydroxide were purchased from Fluka (Buchs, Switzerland). The analytical standard used for the calibration curve, fluorescein thiohydantoin of *N*-(2,3-dihydroxypropyl)valine (diHOPrVal-FTH), was synthesized previously within the research group [15]. Fluorescein isothiocyanate (FITC) was purchased from Karl Industries (Aurora, OH, USA) and potassium hydrogen carbonate (KHCO_3_) from Merck (Darmstadt, Germany). All other chemicals (analytical grade) were obtained from Sigma Aldrich (St Louis, MO, USA).

### 2.2. Study Population

Blood samples from 50 children at the age of about 12 years (35 boys, 15 girls) were obtained from a study in 2014 by the National Food Agency in Sweden regarding food-related exposures in children of school age (reviewed by the Regional Ethical Review Board, Uppsala, Sweden; No. 2013/354 (date of approval: 13 December 2013); following the rules of the Declaration of Helsinki). Venous blood from each individual was sampled at one occasion, and the samples used for they analysis of Hb adducts were centrifuged at 1500× *g* for 10 min to separate red blood cells (RBCs) from the plasma prior to storage of the RBC at −20 °C. In addition to these samples, measurements were also conducted on blood samples from six non-smoking and six smoking adults (males), collected earlier (1997) with ethical approval (from the Regional Ethical Review Board, Stockholm, Sweden; No. 96-312 (date of approval: 14 October 1996). In connection to the blood collection, these blood samples were centrifuged at 1500× *g* for 10 min, to separate RBCs and plasma. The RBCs were washed three times with an equal volume of 0.9% NaCl followed by centrifugation and lysis by the addition of an equal volume of distilled water prior to storage at −20 °C.

### 2.3. Synthesis of Internal Standard

The internal standard (IS), *N*-(2,3-dihydroxypropyl)-(^13^C_5_)valine fluorescein thiohydantoin (FTH), was synthesized in two steps. First, RS-glyceraldehyde (21.6 mg, 240 µmol) and (^13^C_5_)valine (13.7 mg, 116 µmol) were mixed in 3.6 mL methanol, followed by the addition of sodium cyanoborohydride (8.9 mg, 142 µmol) for a reduction of the formed Schiff base. The reaction solution was mixed (750 rpm) for 20 hours at 37 °C, followed by evaporation of the solvent. In the second step, the product, *N*-(2,3-dihydroxypropyl)-(^13^C_5_)valine, was dissolved in 5 mL acetonitrile (40%, aq.) with 0.125 M KHCO_3_ and 540 µL FITC (90 mg, 231 µmol, dissolved in dimethylformamide) to generate the final FTH derivative to be used as IS. The derivatization reaction was kept for 20 hours at 37 °C during mixing (700 rpm), before termination by addition of 1 M HCl (250 µL, 250 µmol). The reaction solution was stored in the freezer overnight followed by centrifugation for 10 min (5000 rpm at 4 °C). 

The supernatant was filtered and concentrated to ca. 2 mL, and the final product to be used as IS was separated from by-products using semi-preparative HPLC-UV. Eight injections of each 250 µL was applied on a Hichrom C18 column (10 mm × 250 mm, 5 µm). The mobile phase started at isocratic mode for 8 min, followed by gradient mode from 40% A (water) and increasing to 100% B (acetonitrile) in 1 min, which was kept for 4 min before re-equilibrating of the column for 3 min prior to the next injection. The flow rate was 4 mL/min and the total run time 16 min. The UV spectrophotometer (Shimadzu SPD-6A) was set to the wavelength (λ) of 274 nm. 

The identity and the purity of the product and the *m/z* was confirmed using LC/UV/MS/MS (API 3200 Q-Trap, AB Sciex, Concord, ON, Canada) running in full scan positive mode (*m*/*z* 80–800). Five µL was injected onto the column (Discovery HS, C18, 150 × 2.1 mm, 3 µm) with a gradient starting from 90% A (water, 0.1% formic acid) for 0.5 min and increasing to 40% B (acetonitrile, 0.1% formic acid) in 6.5 min. A further increase to 100% B was kept for 2 min followed by re-equilibration of the column. The flow rate was 0.2 mL/min and the total run time was 12 min. All settings for the MS instrument were essentially as in previous studies [11,15]. Finally, the solvent of the verified synthesized product was evaporated until dryness, and the yield of the product was determined gravimetrically to be 7.5 mg (13.2 µmol, 11.4%).

### 2.4. Procedure for Hemoglobin Adduct Measurement

The blood samples (250 μL) were prepared for analysis according to the FIRE procedure, where the fluorescein isothiocyanate (FITC) reagent is used for the measurement of adducts (*R*) from electrophilic compounds with a modified Edman procedure [11,12]. The hemoglobin (Hb) content was measured in all samples (RBCs diluted with water, 1:1) prior to derivatization and detachment of N-terminals with adducts with FITC during mixing at 37 °C overnight. Internal standard (*N*-(2,3-dihydroxypropyl)-(^13^C_5_)valine) was added prior to the work-up procedure that was performed as described in several previous papers [15,17,19]. A final sample volume of 100 µL (40% acetonitrile in water) was used for analysis of Hb adducts with ultra-high performance liquid chromatography (UPLC^TM^) and high-resolution mass spectrometry (HRMS) (Section 2.6). The intraday variability of the FIRE procedure was investigated by processing three individual blood samples from the children five times in parallel at one occasion.

Calibration samples were prepared by adding known concentrations of diHOPrVal-FTH to bovine blood (Håtunalab AB, Bro, Sweden) followed by the work-up procedure. Two sets of calibration samples were prepared: Set 1) in duplicates at four levels at 0.04–1.6 pmol/sample (for measurement of background levels in human samples) and Set 2) in triplicates at six levels at 11–600 pmol/sample (for k_val_ determination). The preparation and analysis of the calibration samples were as described by Aasa et al. [15], with the exception of the analysis of the samples from Set 1, which were analyzed with HRMS (described in Section 2.6).

### 2.5. Measurement of Reaction Rate and Calculation of Internal Dose from Adduct Levels

The daily adduct level increment (*a*) was calculated from the measured steady state level of the adducts (*A_ss_*) and the erythrocyte lifetime (*t_er_*), according to Equation (1). The *t_er_* in humans was assumed to be 126 days [13,20,21].

(1) $$A_{ss}=a\;\frac{t_{er}}{2}$$

The daily adduct increment was then used for calculation of daily AUC, by using the second-order rate constant for the reaction between glycidol and the N-terminal valine, k_val_. This constant was determined by incubation of glycidol with fresh human blood from four individuals (from Komponentlab, Karolinska University Hospital, Huddinge, Sweden). Triplicate samples of whole blood from each individual at three dose levels, 0 (control), 125 and 250 μM glycidol (Hb: 102–136 g/L), were incubated for one hour at 37 °C during mixing (750 rpm). The incubations were finalized by centrifugation and washing according to previous procedures [15]. The samples (250 µL) were then derivatized with FITC overnight followed by work-up and analysis by LC/MS/MS, as described by Aasa et al. [15] and as the calibration curve Set 2 (Section 2.4). The k_val_ could then be determined and be used for the calculation of the in vivo doses (AUC) from measured adduct levels (*A*) according to Equation (2), assuming that glycidol at these concentrations is stable during the 1-hour incubation time, as observed earlier [15]. For calculation of the daily AUC the *A* is replaced with the daily adduct increment (*a*) calculated in Equation 1.

(2) $$AUC\;\left(µMh\right)=\;\frac{A\;\left(pmol/g\;Hb\right)}{\;k_{val}\;\left(pmol/g\;Hb/\mu Mh\right)}$$

### 2.6. LC/MS/MS System

The analysis of Hb adducts in the in vitro incubations was performed with a triple quadrupole LC/MS/MS instrument, as described by Aasa et al. [15]. Compared to the previous analysis, the *m/z* transitions 563.1 > 447.1 (from glycidol) and 565.1 > 449.1 (from the internal standard) were also included in the analysis. 

The analysis of Hb adducts in blood samples from the studied groups of humans was conducted with a Dionex Ultimate 3000 UHPLC system connected to an Q ExactiveTM HF Hybrid Quadrupole-Orbitrap™ high-resolution mass spectrometer (HRMS) (Thermo Fisher Scientific, MA, USA). The chromatographic separation was performed by injection (20 μL) on an Acquity UPLC^TM^ HSS C18 column, 2.1 × 100 mm, 1.8 μm (Waters, Sollentuna, Sweden). The mobile phase (0.3 mL/min) was running in gradient mode, starting at 80% A (0.1% formic acid in H_2_O:ACN; 95:5, *v*/*v*) and increasing to 100% B (0.1% formic acid in H_2_O:ACN; 5:95, *v*/*v*) in 9 min. The final composition was kept for 2.5 min before re-equilibration for 3 min. *N*-(2,3-Dihydroxypropyl)valine fluorescein thiohydantoin was monitored in parallel reaction monitoring (PRM) mode with the resolution 60 000 and the normalized collision energy (NCE) on 45. The software XCalibur (Thermo Fisher Scientific, MA, USA) was used for processing of the data. The levels of the Hb adduct was quantified by using the accurate masses *m*/*z* 563.1463 (diHOPrVal-FTH) and *m*/*z* 568.1629 (internal standard, *N*-(2,3-dihydroxypropyl)-(^13^C_5_)valine fluorescein thiohydantoin), and the specific fragments associated with diHOPrVal-FTH; *m/z* 503.0893, 460.0704 and 447.0629 and the internal standard; *m/z* 505.0955, 462.0772 and 449.0694, with a 3–5 ppm mass tolerance.

### 2.7. Statistical Analysis

For the measured and calculated parameters (Hb adduct levels, AUC and intakes), the minimum and maximum values along with the mean values and the standard deviations are reported for the studied groups. A *t*-test and the Grubb´s test were used for the analysis of differences between the studied groups and for testing of outliers, respectively.

## 3. Results 

**Figure 2:**
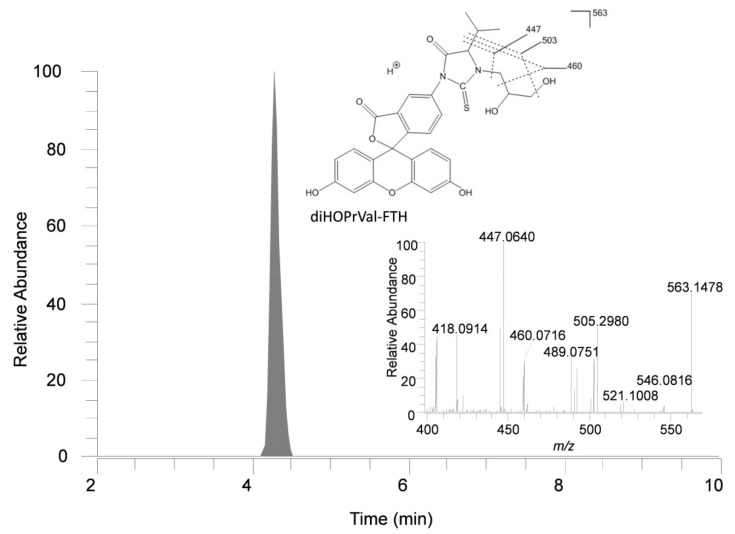
Ion chromatogram from a human sample and exact mass spectrum for the fluorescein thiohydantoin (FTH) derivative (diHOPrVal-FTH) of the N-terminal valine adduct formed by glycidol (6.6 pmol/g Hb at 3 ppm mass tolerance). The *m/z* fragments 503, 460 and 447 were used for quantification of the adduct diHOPrVal-FTH (*m/z* 563).

For the present study we used high resolution mass spectrometry (HRMS) operating in parallel reaction monitoring (PMR) mode, which improved the selectivity for detection of the adduct *N*-(2,3-dihydroxypropyl)valine (diHOPrVal). We also synthesized an isotopically substituted internal standard, *N*-(2,3-dihydroxypropyl)-(^13^C_5_)valine fluorescein thiohydantoin, specific for the quantification of the studied adduct, which is an improvement for the quantification compared to our previous studies [15,18]. A representative example of a chromatogram from analysis of a human blood sample and a *m/z* spectrum of *N*-(2,3-dihydroxypropyl)valine-FTH is shown in Figure 2. The variability of the FIRE method was tested by processing five parallel blood samples from three children, which showed a relative standard deviation of 4.2–7.3% of the replicates (data not shown).

**Figure 3:**
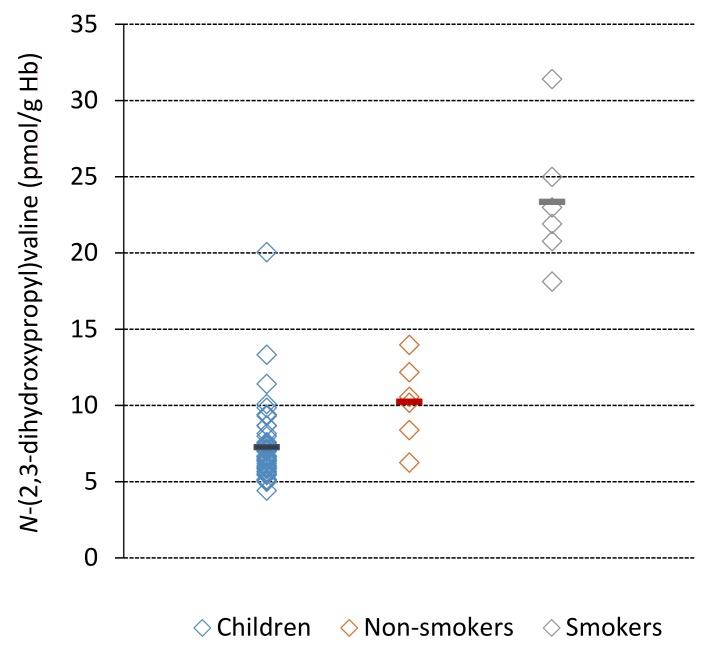
*N*-(2,3-Dihydroxypropyl)valine adduct levels in blood from children (*n* = 50) and, non-smoking (*n* = 6) and smoking (*n* = 6) adults. The mean values are marked as horizontal bars. The higher level observed in the smokers is likely due to presence of glycidol in tobacco smoke (see Section 4).

The Hb adduct was possible to quantify in all studied subjects. The adduct levels observed in the samples from the children (*n* = 50) varied between 4.4 and 20 pmol/g Hb (Figure 3, Table 1A). No statistically significant difference in the levels was observed between the sexes of the children. For the adults, the range of the observed adduct levels was 6.3–31 pmol/g Hb (Figure 3, Table 1A). Although this group consisted of a small number of subjects, there was a statistically significant difference of the adduct levels between the smoking and the non-smoking subjects (adults), with about twice higher mean levels in the smokers (23.4 pmol/g Hb) compared to the non-smokers (10.3 pmol/g Hb) (*p* < 0.01). The adduct levels of the non-smoking adults were in the range of the adduct levels in the children (Figure 3, Table 1A). Assuming that a chronic exposure (of glycidol or its precursor) is giving rise to the observed adduct levels, the daily adduct increment was calculated using Equation 1 (Table 1A). 

The daily intake of glycidol (µg/kg per day) was in the next step calculated from the obtained daily Hb adduct level increments. To perform this calculation, the relation between the adduct level (or in vivo dose) and administered dose of glycidol is required. We used the results recently published by Abraham et al., who studied the relation between glycidol-induced diHOPrVal adduct levels and administered dose of glycidol from palm fat in human subjects [22]. The adduct increment in their study was calculated to be 82 pmol/g Hb per mg glycidol/kg body weight (b.w.). This figure was used to estimate the mean daily intakes of glycidol in the different groups of subjects in our study, as presented in Table 1B.

**Figure 4:**
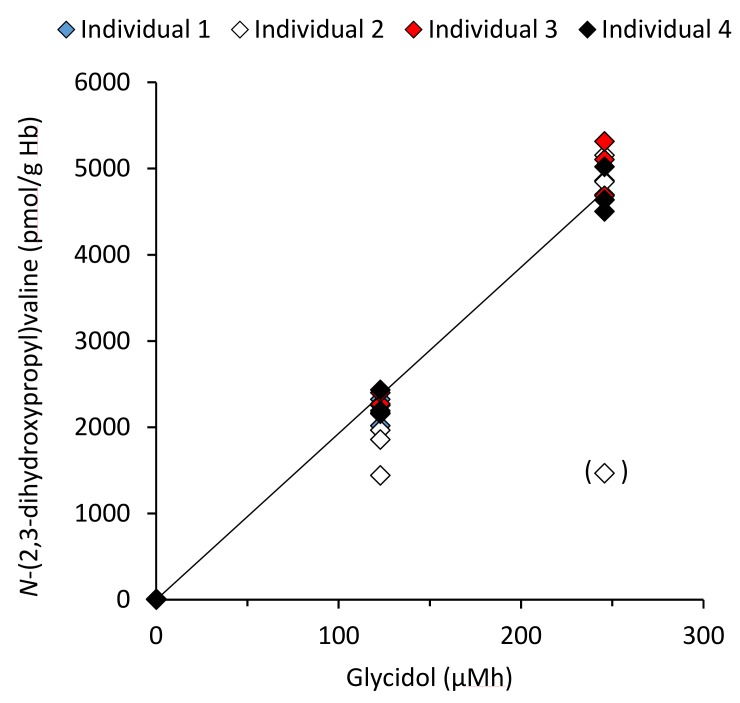
Determination of the second-order reaction rate constant, k_val_, for the formation of the *N*-(2,3-dihydroxypropyl)valine adduct in Hb. The data points represent the adduct levels from 1-hour incubations of glycidol with fresh human blood from triplicate samples at each dose level from four individuals, where each point corresponds to the mean from two *m/z* transitions. One replicate at the highest dose for individual 2 (data point in parenthesis) was excluded in the analyses as it was judged as an outlier (Grubbs test).

Further, the daily in vivo doses (AUC) of glycidol in the studied human individuals (Table 1B) were calculated from the daily adduct level increments and the k_val_ according to Equation 2. The second-order reaction rate constant (k_val_) was determined to be 19.2 ± 0.6 pmol/g Hb per µMh from the linear slopes of plots of the Hb adduct levels (*y*-axis) obtained from triplicate incubations of glycidol at two doses (AUC in vitro: *x*-axis) in human fresh whole blood from four individuals (Figure 4). The AUC was used for the calculation of the cancer risk due to glycidol exposure, further discussed in the Discussion part.

## 4. Discussion

### 4.1. N-(2,3-dihydroxypropyl)valine Adduct Levels

In this study, we quantified the levels of the *N*-(2,3-dihydroxypropyl)valine adduct (diHOPrVal) in samples from 50 children of about 12 years of age, showing a fivefold variation in the adduct levels (ca. 4–20 pmol/g Hb). No significant difference was observed between the sexes of the children. The diHOPrVal adduct levels were also quantified in a small number of adults (*n* = 12), where the mean adduct levels where about the double in the smokers compared to the non-smokers. As glycidol is known to be present in tobacco smoke, this was expected [23,24]. The mean Hb adduct level in studied non-smoking adults indicated approximately the same exposure for this group as for the children. A larger sample size from adults should be included for a more reliable comparison.

The diHOPrVal adduct in Hb has earlier been quantified only in small groups of adults in a few published studies, which all show somewhat lower levels compared to our study (Table 2). The observed variation of the mean adduct levels between the different studies may be due to differences in exposure between the studied groups, but also due to the fact that the analyses are performed at different laboratories and with different analytical methods for measurement of the N-terminal adduct in Hb; the *N*-alkyl Edman (GC/MS/MS) and the FIRE procedure (LC/MS/MS), and with no inter-calibration between the laboratories.

**Figure 5:**
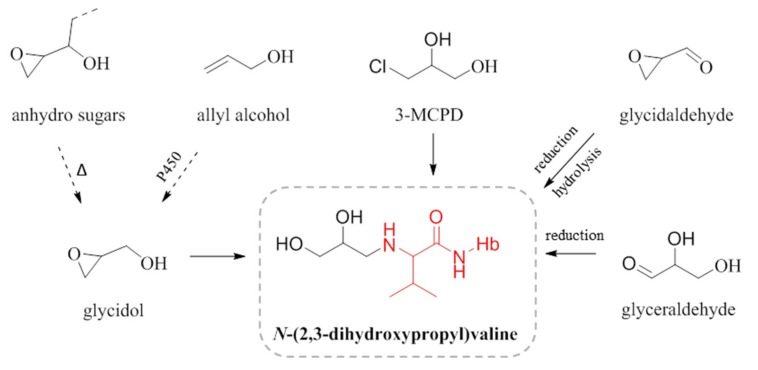
Examples of possible precursors to *N*-(2,3-dihydroxypropyl)valine c.f. [29,30].

We have assumed that the observed diHOPrVal adduct levels in children and non-smokers originate from the exposure to the genotoxic compound glycidol via food, but this adduct may also theoretically originate from other precursors (Figure 5). One possibility is the food contaminant 3-monochloropropane-1,2-diol (3-MCPD), often occurring in parallel with glycidol in food. 3-MCPD would however give a very small contribution, as 3-MCPD has more than 1000 times lower rate constant for formation of the adduct to the N-terminal in Hb compared to glycidol [28]. The food-related compounds allyl alcohol, found in garlic, or anhydro sugars from carbohydrates, can also theoretically be precursors to the adduct [29,30]. The formation of glycidol from allyl alcohol could be assumed to be possible via a metabolic oxidation. The heating of carbohydrate-rich food (anhydro sugars) could theoretically form glycidol, which was indicated in an animal experiment with feeding with heat-processed feed and measurement of the diHOPrVal adduct [30]. Other theoretically potential precursors are the endogenously produced glyceraldehyde and glycidaldehyde. Both compounds, though, require reduction after formation of a Schiff base to the N-terminal valine in Hb to form the stable diHOPrVal adduct [30]. It is not known whether the reduction of protein adducts from Schiff bases occurs in vivo, but it was observed to occur in blood in vitro [31]. Epichlorohydrin, from occupational exposure, also could form diHOPrVal but it is not a probable exposure source in the presently studied group of humans [27]. Thus, other sources to the measured adduct than glycidol cannot be excluded, which could potentially lead to an overestimation of the in vivo doses (AUC) of glycidol in the studied subjects. It is obvious that low molecular mass adducts in many cases could have several possible precursor electrophiles.

In this study, we calculated the AUC of glycidol from the quantified diHOPrVal levels in human subjects, assuming that glycidol is the dominating source of the adduct. The differences in the measured adduct levels and the corresponding intakes and AUC of glycidol (fivefold) between all children (Table 1) could partly be explained by different dietary habits, as glycidyl fatty acid esters are present in different types of food products [3], but also by different genotypes/phenotypes for metabolizing enzymes (e.g., epoxide hydrolase and glutathione transferase, c.f [3]). Furthermore, we could not observe any significant correlations between the diHOPrVal levels in the children and any type of registered food product (from food frequency questionnaires) with potential impact on glycidol exposure (bread, sweets, chips and other fried food) in this limited study (data not shown). Studies of a larger number of subjects and using food frequency questionnaires with more specific questions could possibly enable a sufficient statistical material for such analyses.

### 4.2. In vivo dose and Intake of Glycidol

Knowledge about the AUC of a particular exposure gives the possibility for a more accurate estimation of the cancer risk. Assuming that the AUCs and the intakes calculated for the studied subjects reflect the exposure to the general European population, the values imply a higher exposure to glycidol than expected, from the mean glycidol intakes estimated by the European Food Safety Authority (EFSA); ca. 0.2 µg/kg b.w./day and 0.6 µg/kg b.w./day for adults and children, respectively [3] and the Swedish National Food Agency (NFA); 0.1 µg/kg b.w./day for adults [32] (Table 3). The relation between adduct level and intake of glycidol in humans was obtained from a recently published human exposure study by Abraham et al., which involved a good number of persons (11) with intake of palm fat oil over 4 weeks corresponding to a mean daily intake of glycidol of 4.3 µg/kg b.w. [22]. The methods for measurement of the diHOPrVal adducts used by Abraham et al. and by us are not inter-calibrated, which might contribute to some uncertainty in this calculation, just like the figure on a mean lifetime of erythrocytes of 126 days (cf., Mitlyng et al. [33] and Abraham et al. [22]).

The corresponding figures of the AUC per administered dose of glycidol, obtained by different methods, are ca. 35% lower both for rats and monkeys [34,35] compared with the value calculated for humans, which indicates that there are no major differences in the disappearance rate of glycidol between these species and which also supports the reliability of the obtained human data.

### 4.3. Human Cancer Risk

Different models have been used to assess the human cancer risk of glycidol based on published carcinogenicity data from studies in rodents [36,37]. The European Food and Safety Authority (EFSA) has used the Margin of Exposure (MOE) approach, which is based on estimated intake values of glycidol and the reference point T25 (the dose corresponding to a 25% tumor incidence in the animals used in the carcinogenicity studies) [3]. Using the MOE, EFSA concluded that there is a health concern associated with glycidol exposure. Furthermore, the California Environmental Protection Agency (C. EPA) has calculated a non-significant risk level (NSRL; 1 cancer case in 10^5^ individuals over life-time) of 0.54 µg glycidol per day using an additive risk model [38]. 

Common for these two models are that the estimation of the cancer risk is based on extrapolations from rat to human and the administered doses of glycidol in the carcinogenicity studies. As an improvement of cancer risk estimations, our group at Stockholm University has developed a risk model based on species extrapolation via internal doses (AUC) and background tumor incidence. This model is referred to as the multiplicative risk model and has recently been validated for glycidol, presented in a forthcoming paper [33] and a few other genotoxic carcinogens [39,40,41]. With this model, described in detail in the referred papers, a cancer risk coefficient (β), which describes the relative tumor risk per in vivo dose, is derived. The relative risk coefficient has been shown to be approximately independent of tumor site, sex, and species for all tested compounds as well as for ionizing radiation. Thus, a common risk coefficient can be derived from the responding sites in the test species in animal cancer tests with a compound. Accordingly, this risk coefficient is assumed to also be valid in humans and can be used for the calculation of the human cancer risk for the studied specific exposure, when in vivo dose and background cancer incidence is known.

For the calculation of the human cancer risk due to glycidol exposure, the in vivo dose over a lifetime (70 years) is compared to the obtained cancer risk coefficient β [33], which is somewhat higher than the risk coefficient obtained by an additive model, as by C.EPA [38]. In the present work, we estimated a mean daily intake of 1.4 μg glycidol per kg bodyweight for the children, which implies about 36 mg/kg during a lifetime (70 years). This is equivalent to the cumulative AUC of ca. 150 μMh (Table 3). Assuming that the background tumor frequency in the general human population is ca. 30% [42], the given lifetime exposure condition lead to an estimate of ca. 200 additional cancer cases in a population of 100,000 (i.e., relative risk increment) at the given exposure condition. In the calculations, we have not considered different contribution to the risk from exposure at different ages, where children in general have higher risk increments per AUC compared with adults. This was observed for subjects exposed to ionizing radiation where the excess relative risk for children is higher compared to adults, as reviewed by Kutanzi et al. [43]. 

## 5. Conclusions

This is the first study measuring the Hb adduct *N*-(2,3-dihydroxypropyl)valine and calculation of the corresponding in vivo doses of glycidol in children. The observed variation in the in vivo doses of glycidol within the children´s cohort is likely due to dietary habits and/or different genotypes/phenotypes of metabolic enzymes. The data on diHOPrVal adduct levels in the children as well as in the small group of adults, despite some remaining uncertainties, indicate that calculated intakes of glycidol give contributions that exceed what is considered to be an acceptable cancer risk, using a multiplicative cancer risk model. The obtained data, calculated intakes, and corresponding estimated cancer risks emphasize the importance of further clarifying the background exposure to glycidol from food, as well as possible other sources to the observed diHOPrVal adduct levels in the population, particularly in children.

## Figures and Tables

**Table 1:** (A) Measured steady state Hb adduct levels (*N*-(2,3-dihydroxypropyl)valine) in blood samples from children and adults and corresponding calculated daily adduct level increments, and (B) the corresponding estimated daily in vivo doses (AUC) and intakes of glycidol.

Group	A			B	
	Hb Adduct (pmol/g Hb)		Daily Hb Adduct Level Increment (pmol/g Hb)	Daily AUC ^b^ (nMh)	Daily Intake (µg/kg/day)
Subjects (*n*)	Mean ^a^ ± SD	Min–Max	Mean ^a^ ± SD	Mean ± SD	Mean ± SD
**All children (50)**	7.3 ± 2.5	4.4–20.1	0.11 ± 0.04	6.0 ± 2.1	1.4 ± 0.5
**Boys (35) ^c^**	7.2 ± 1.8	4.4–13.3	-	-	-
**Girls (15) ^c^**	7.4 ± 3.7	5.0–20.1	-	-	-
**Non-smokers (6)**	10.3 ± 2.7	6.3–14.0	0.16 ± 0.04	8.5 ± 2.3	2.0 ± 0.5
**Smokers (6) ^d^**	23.4 ± 4.6	18.1–31.4	0.37 ± 0.07	19.3 ± 3.8	4.5 ± 0.9

^a^ Mean levels quantified from four different *m/z* transitions in the MS analysis of the studied Hb adduct. ^b^ The daily AUC was calculated from the daily adduct level increment (Equation 1) and the second-order reaction rate constant, k_val_ (Equation 2), assumed to be at steady state (from chronic exposure). ^c^ The calculations of the daily Hb adduct increment, AUC and intake have been reported for boys and girls together as no statistical differences were observed between the sexes. ^d^ Glycidol in tobacco smoking is likely contributing to the daily intake level (see Section 4).

**Table 2:** Steady state levels of the Hb adduct *N*-(2,3-dihydroxypropyl)valine (diHOPrVal) in adult subjects (from different countries) without any known exposure, obtained in published studies.

Studied Group	No. of Subjects	diHOPrVal (pmol/g globin)	Analytical Method	Reference
**Non-smokers**	11	4.1 ± 0.8 ^a^	LC/MS/MS	[22]
**Non-smokers**	12	3.3 ± 0.8 ^a^	LC/MS/MS	[25]
**Non-smokers**	6	7.1 ± 3.1 ^b^	GC/MS/MS	[26]
**Non-smokers**	3	6.8 ± 3.2 ^b^	GC/MS/MS	[27]
**Non-smokers**	4	2.1 ± 1.1 ^b^	GC/MS/MS	[27]
**Non-smokers**	6	10.3 ± 2.7 ^a^	LC/MS/MS	present study
**Smokers**	6	13.1 ± 12.4 ^b^	GC/MS/MS	[27]
**Smokers**	6	9.5 ± 2.2 ^b^	GC/MS/MS	[27]
**Smokers**	6	23.4 ± 4.6 ^a^	LC/MS/MS	present study

^a^ FIRE procedure (adduct level expressed as per g Hb, approximately the same as per globin). ^b^ *N*-alkyl Edman method.

**Table 3:** Estimated daily intakes of glycidol and calculated AUC at corresponding estimated lifetime exposures of glycidol, calculated from Hb adduct levels measured in children and adults (non-smokers) in the present study. Daily intakes estimated from dietary patterns by the European Food Safety Authority (EFSA) and the Swedish National Food Agency (NFA) are used for comparison [3,32].

Studied Group	Number of Subjects	Daily Intake (μg/kg b.w./day) Mean [Min–Max]	Estimated Lifetime AUC (µMh) Mean (Approximately)
Present study			
Children	50	1.4 [0.9–3.9]	150
Adults (non-smokers)	6	2.0 [1.2–2.7]	230
Intake estimated by authorities			
Children (EFSA)	n.a.	0.6 [0.4–0.9]	-
Adults (EFSA)	n.a.	0.2 [0.2–0.3]	-
Adults (NFA)	n.a.	0.1	-

^a^ n.a.: not available.
